# Left Ventricular Assist Devices 101: Shared Care for General Cardiologists and Primary Care

**DOI:** 10.3390/jcm8101720

**Published:** 2019-10-18

**Authors:** Aditi Singhvi, Barry Trachtenberg

**Affiliations:** Houston Methodist DeBakey Heart & Vascular Center 6550 Fannin Street, Houston, TX 77030, USA; btrachtenberg@houstonmethodist.org

**Keywords:** left ventricular assist devices, management, complications, outcomes

## Abstract

Ambulatory patients with a left ventricular assist device (LVAD) are increasing in number, and so is their life expectancy. Thus, there is an increasing need for care of these patients by non-LVAD specialists, such as providers in the emergency department, urgent care centers, community-based hospitals, outpatient clinics, etc. Non-LVAD specialists will increasingly come across LVAD patients and should be equipped with the knowledge and skills to provide initial assessment and management for these complex patients. These encounters may be for LVAD-related or unrelated issues. However, there are limited data and guidelines to assist non-LVAD specialists in caring for these complex patients. The aim of our review, targeting primary care providers (both inpatient and outpatient), general cardiologists, and other providers is to describe the current status of durable LVAD therapy in adults, patient selection, management strategies, complications and to summarize current outcome data.

## 1. Introduction

Heart failure (HF) affects an estimated 6.5 million people in the United States currently. Optimal medical [1,2] and resynchronization device [3] therapy can lead to reverse myocardial remodeling with symptomatic and survival benefit. However, about 0.5%–5% of patients with HF will progress to end-stage disease [4]. These patients have a poor quality of life with recurrent hospitalizations and a high mortality rate (1-year mortality in ambulatory class III–IV patients is >25% and exceeds 50% in class IV patients) [5,6]. Therapeutic options for these patients include heart transplant or left ventricular assist device (LVAD) therapy, either as a strategy of a bridge to transplantation (BTT) or as destination therapy (DT). Although heart transplant remains the established therapy of choice for these patients, it is limited by organ availability, wait list mortality, and other comorbidities, such as fixed pulmonary vascular resistance [7]. Fortunately, LVADs to support patients with end-stage HF with reduced ejection fraction (HFrEF) have continued to evolve, with improvement in technology, durability and miniaturization. These devices are a life-saving option for advanced HF patients who are either not candidates for a heart transplant or too high-risk to safely wait for a transplant on medical therapy alone. The International Society for Heart and Lung Transplantation (ISHLT) reported that 19.1% of transplant recipients were bridged with a mechanical support device in 2000, and this number increased to 41.0% in 2012 [8]. LVADs can also be a bridge to decision for patients who are not candidates for transplant at the time of implantation but may become suitable candidates after the procedure [9] and may also be utilized to promote myocardial recovery in a bridge to recovery strategy. Outcomes post-LVAD implantation have continued to improve, with 1-month survival estimates at 96%, and 1- and 2-year survival estimates at 83% and 73%, respectively [10].

Over the past decade, the use of LVADs has increased significantly. In the United States, approximately 421 isolated LVAD implants were performed in 2008. A total of 2118 LVADs were implanted in 2017, which is a significant increase from only 459 implants in 2008. There was a steady increase in the number of LVADs implanted from 2008 to 2015, peaking at 2754 devices annually. Since 2016, the INTERMACS Database reported a decline in annual LVAD volume, related in part to a number of patients who received non-FDA-approved pumps as part of the MOMENTUM 3 clinical trial or a delay in enrollment of patients during contractual negotiations in 2017 between STS and participating hospitals [10]. So far, more than 19,000 patients have received a continuous-flow LVAD in the United States [10]. Thus, there is an increasing need for care of these patients by non-LVAD specialists, such as providers in the emergency department, urgent care centers, outpatient clinics, etc. These encounters may be for LVAD-related or unrelated issues. However, there is limited literature to assist non-LVAD specialists in caring for these complex patients. The aim of this state-of-the-art review, targeting internists and general cardiologists, is to describe the current status of durable LVAD therapy in adults, patient selection, management strategies, complications and to summarize current outcome data.

### Evolution of LVAD Therapy 

In 1963, Drs. Michael DeBakey and Domingo Liotta implanted the first LVAD in a patient who suffered a cardiac arrest after an aortic valve replacement surgery [11]. The patient died on the fourth postoperative day, attributed to anoxic encephalopathy.

First-generation pulsatile LVADs were volume-displacement pumps, using a diaphragm and unidirectional valves to mimic the pulsatile cardiac cycle through diastolic filling and systolic emptying of the device. However, these devices were large, noisy and had limited durability due to multiple mechanical parts that were subject to wear and tear [12].

To overcome the limitations of pulsatile-flow (PF)-LVADs with respect to mechanical wear and to permit application to smaller recipients, the field focused on development of continuous-flow (CF) LVADs. CF-LVADs have a smaller profile with a single moving part (i.e., impeller/rotor). They have significantly improved durability, reliability, reduced noise emission and reduced incidence of adverse events. There are currently three such durable (i.e., able to be discharged home) LVADs approved for adults by the U.S. Food and Drug Administration (FDA): the HeartMate II (Abbott Laboratories, Abbott Park, IL, USA), the HeartWare ventricular assist device system (HVAD) (Medtronic, St. Paul, MN, USA), and the HeartMate 3 (St. Jude Medical, Pleasanton, CA, USA).

## 2. Engineering and Pump Technology

### Design and Function

**Figure 1:**
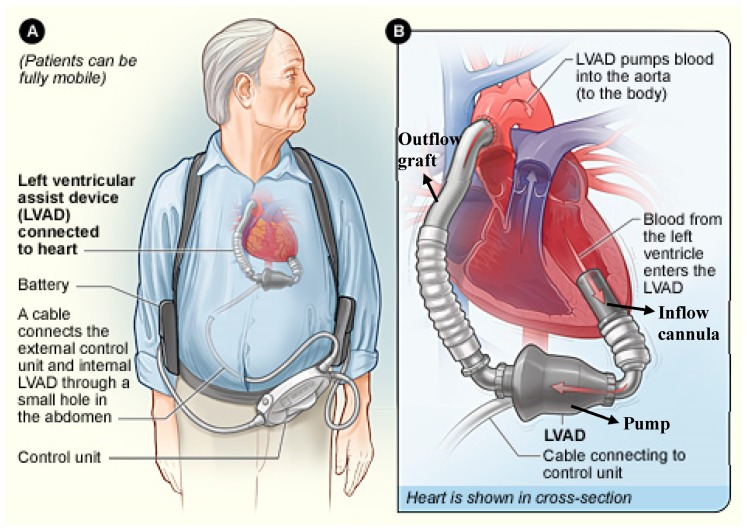
Components of a left ventricular assist device system. (**A**): Representation of a patient with an LVAD depicting its various external components. (**B**): Inset showing the heart in cross-section with the internal components and connections of an LVAD.

**Figure 2:**
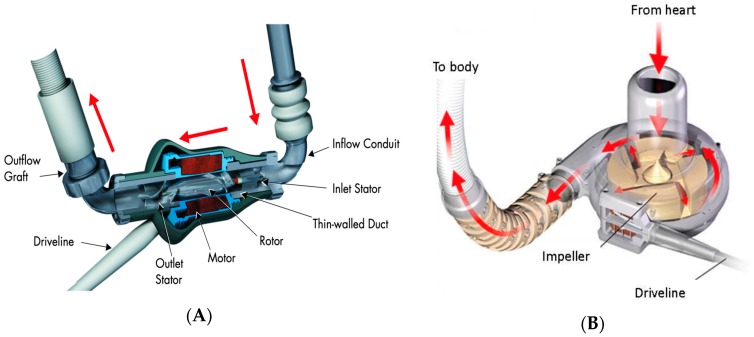
Pump housing and impeller design. (**A**): (left) Axial flow pump: HeartMate II LVAD (taken from Figure 2 in the HeartMate II left ventricular assist system instructions for use). *(***B**) (right) Centrifugal flow pump: HeartWare assist device (taken from Figure 2 in the HeartWare assist device patient manual).

The contemporary LVADs are all continuous-flow pumps. They vary in design but have the same basic components. The components of an LVAD system are depicted in Figure 1. Blood from the left ventricle enters the pump through an inflow cannula which is surgically implanted into the left ventricular apex. The pump contains a rotor or impeller which propels blood. The CF pumps are of two varieties based on the type of impeller: axial flow and centrifugal flow. The outflow of the impeller is parallel to the axis of rotation in an axial flow pump. The rotor spins on mechanical bearings in the HeartMate II device. The rotating element propels blood forward (Figure 2A). In centrifugal pumps, the impeller outflow is directed perpendicular to the axis of rotation (i.e., HVAD and HeartMate III). The motor is like a rotating disk with blades that push the blood from impeller blades to the outflow cannula along a tangential course (Figure 2B). Blood is returned from the pump to the ascending aorta via an outflow graft. 

A surgically tunneled cable, called a driveline, connects the pump to an external control unit, which operates and monitors the function of the pump. The driveline usually exits the body through the abdominal wall. The external controller is connected to a power source, which may be batteries; or a power module which is plugged into an AC wall source when the batteries are charging. Batteries usually last up to 12 h, allowing the patient to ambulate freely untethered. The remaining battery life is displayed on the external controller. The external controller also displays other parameters describing pump function, such as the LVAD speed, flow, power and pulsatility index in the Heartmate devices (explained in detail below).

Most pumps are now designed to be contact-free, without mechanical bearings and an impeller suspended using magnetic and/or hydrodynamic systems. The presence of mechanical bearings is thought to predispose to formation of thrombi on the metal. Thrombus formation leads to further propagation of the thrombus by leading to friction and, therefore, increasing heat and turbulence. The pump in the HVAD device uses a layer of blood to lift the rotor, known as hydrodynamic levitation. In the Heartmate 3, the motor is suspended magnetically without any mechanical bearings.

In addition to being frictionless due to the absence of mechanical bearings, the HeartMate 3 LVAD also has wide blood-flow passages to reduce shear stress and an intrinsic pulse (~20/min) by transiently decreasing the impeller speed. These design changes are engineered to reduce stasis, minimize destruction of red blood cells, avert thrombosis, and thus improve hemocompatibility. Current trends in the evolving technology of LVADs are focusing on generating pulsatility, further miniaturization, total implantability and remote monitoring.

Present day LVADs, both axial and centrifugal, continuously empty blood from the LV into the systemic circulation. The rotor or impeller within the pump propels blood by spinning at a high speed. Blood flow is proportional to the pump speed, though the relationship is not linear. The speed at which the LVAD pump operates, is essentially the only parameter that can be set by the provider. The LVAD speed is selected at rest to ensure adequate left ventricular unloading. Inadequate LV unloading may result in pulmonary edema and heart failure, while excessive unloading could lead to what are called suction events, resulting in hypotension and arrhythmias. Blood flow through the LVAD is proportional to the motor current or power consumption. Therefore, the blood flow is estimated based on the power consumption and is also displayed on the external controller. However, since it is an estimated value, it may be inaccurate, especially in cases of hemodynamic derangements, such as aortic insufficiency or pump thrombosis.

Continuous-flow pumps do not typically generate a pulse; however, the interaction with the native ventricular function leads to phasic changes in blood flow through the LVAD. This fluctuation is termed pulsatility index for the HeartMate devices and is displayed as a waveform for HVAD. Typical values for pump parameters are mentioned in Table 1. The key differences between the three current LVADs in terms of design and function are summarized in Table 2.

## 3. Patient Selection and Outcomes

While patients are still distinguished by upfront strategy of BTT or DT, there is significant dual crossover that occurs once they are on LVAD therapy. For example, some patients may develop contraindications to transplantation while waiting for transplant on LVAD support. Conversely, many patients have co-morbidities that may be reversible (i.e., “fixed” pulmonary hypertension, acute renal injury) after a more prolonged period of hemodynamic support with an LVAD. In addition, many patients eligible for transplantation simply never receive an organ because of the relative shortage of suitable donors. These issues have recently raised the question of whether categorization as BTT or DT remains clinically meaningful.

Indications for durable mechanical support are generally derived from inclusion criteria used in the clinical trials leading to FDA approval. The criteria generally reflect end-stage heart failure, but are somewhat subjective. These criteria include:

1. LVEF < 25%
2. NYHA IIIb–IV symptoms for at least 45 of the last 60 days
3. Refractory heart failure symptoms despite optimal medical and device therapy
4. Peak VO2 < 14 mL/kg/min
5. Continued need for IV inotropic therapy due to symptomatic hypotension, worsening end organ function, or persistent pulmonary edema
6. IV inotropic medication use for ≥14 days
7. Intra-aortic balloon pump support for ≥7 days

Recent cancer is not an absolute contraindication for LVAD implant, unlike for heart transplantation. Psychosocial evaluation is important for selection of candidates who may achieve good outcomes. Studies have documented that social network and even marital status are important factors in achieving good outcomes with LVAD therapy [17].

The challenge is in identifying patients who have advanced heart failure, but not decompensated to the degree of declining despite inotropic and/or temporary mechanical support. Early referral is key to ensuring good outcomes post durable mechanical support.

Long-term outcomes for patients on LVAD support continue to improve. The most recent INTERMACS data reports overall survival for patients on CF-LVAD at 1, 3 and 5 years of 83%, 63%, and 46%, respectively [18]. More contemporary data from the MOMENTUM 3 trial show an even better overall survival of 79% at 2 years with HeartMate 3 (compared with 76.7% with HeartMate II) and survival free from disabling stroke or reoperation to replace/remove a malfunctioning device of 74.7% at 2 years [19]. A summary of the key LVAD clinical trials and their reported outcomes is provided in Table 3.

## 4. Shared Care for Patients with LVADs 

As the volume of LVAD patients has increased, a model of “shared care” has emerged, wherein care of the LVAD patients is shared between the implanting center and a community-based, non-implanting site [28,29]. This can reduce the clinical burden for implanting centers, allowing more complex care to be centralized there, while more routine care can be delivered safely at sites in the community. The greatest advantage is probably for the patients who can receive quality care locally, which reduces the financial burden and inconvenience related to traveling to the implanting center. Programs at shared care sites usually consist of a cardiologist with heart failure expertise and advanced practice providers with heart failure training. Education and training of personnel at the shared care site by the implanting center is required. The shared care site also needs LVAD-specific equipment, such as system monitors, batteries, power cables, controllers, dressing supplies. Communication between the implanting site and shared care site is essential to ensure the success of such a model. The shared care site should be provided with all LVAD-related institutional policies and protocols. Patient-related information also needs to be shared, ideally at the time of each visit, so there is no fragmentation in the care of the patient. 

At this time, the greatest value of such a model is in providing routine, ambulatory care. This includes routine LVAD care, assessing device function, driveline evaluation, anticoagulation management, routine laboratory testing and cardiac rehabilitation. Non-LVAD related medical problems, such as diabetes, can also be managed at these sites. Though the implanting center will remain the primary contact for emergencies, it may become necessary for the patient to be stabilized locally prior to transfer. In such cases, the shared care sites can be extremely valuable due to their knowledge of LVADs and of the patient’s history. The implanting center should be contacted as soon as possible in case of emergencies and management should be done in consultation with an LVAD specialist. 

## 5. Long-Term Management

### 5.1. Patient Assessment

#### 5.1.1. History and Physical Examination

Unique aspects of the patient evaluation include assessment for normal LVAD function, and for common (sometimes occult) complications.

The history should include recent device parameters and alarms, symptoms of driveline infection such as discharge and redness, heart failure symptoms, ICD shocks, and signs of hemoglobinuria (e.g., dark urine) that could herald development of LVAD thrombosis. History of overt bleeding, particularly melena, should be elicited, because bleeding from arteriovenous malformations in the gastrointestinal tract is a frequent complication.

Continuous flow devices greatly alter the physical exam in supported patients. This can be challenging to clinicians and first responders in assessing these patients. Patients supported by a CF-LVAD frequently have no palpable pulse (or sometimes faint and intermittent pulse) and blood pressure (BP) may not be measurable by auscultation. Heart sounds are obscured by the hum of the device. Additionally, high placement of the external driveline can impede examination of the liver and assessment of hepatic congestion.

Underlying heart rate and rhythm are best assessed by electrocardiogram (ECG) or telemetry. BP is best estimated manually using a Doppler ultrasound probe and sphygmomanometer (generally brachial) [30]. A Doppler probe is used to auscultate the brachial artery flow in the antecubital fossa. A BP cuff is inflated to pressure about 20 mmHg above the pressure at which the brachial artery flow is occluded. As the cuff is deflated, the pressure at which the brachial artery flow returns is noted as the opening or Doppler pressure. If a patient has a strong, consistent pulse, then the opening pressure likely represents the systolic BP. However, if a patient has minimal or inconsistent pulsatility, then the opening pressure is more likely an estimate of the mean arterial pressure (MAP). The typical MAP target is 60–80 mm Hg, avoiding higher pressures which may increase LVAD afterload and thus decrease forward flow, as well as to decrease the risk of stroke [31]. MAPs above 90 mmHg warrant therapeutic intervention. Newer devices for BP measurement such as the Terumo Elemano BP monitor are also clinically useful [32]. The Elemano device uses double-cuff oscillometric slow deflation technology with results correlating well with the arterial line BP with minimal underestimation of systolic pressure (on average 0.3 mmHg lower) [33]. The device is easy to use and can be used for home blood pressure monitoring. In the EDURANCE Supplemental Trial, patients with a palpable pulse were provided with the Terumo Elemano automated cuff for BP measurement. Patients without a palpable pulse were taught to measure their BP with a Doppler probe and manual cuff. Their goals were, MAP ≤ 85 mmHg with the automated cuff and opening pressure ≤ 90 mmHg with the Doppler probe. With strict BP management, there was a reduction in the overall stroke rate at 12 months, from 22.3% in ENDURANCE to 16.9% in the Supplemental Trial, with a 50% reduction in the hemorrhagic stroke rate [34].

On cardiac auscultation, the “hum” of an LVAD should be appreciated, which is important if there is any concern for pump dysfunction, since the hum can vary depending on pump stress or pump stoppage. 

Another important element of the physical exam for specialists (or for shared care centers) in mechanical device patients is assessment of the percutaneous driveline or lead for infection or damage. If there is a high level of suspicion for infection and LVAD specialists are not available, direct inspection of the driveline site for erythema, swelling, and/or purulent discharge can be performed. This should be performed wearing masks and sterile gloves to avoid transmitting infections to the percutaneous driveline site. It is important to stabilize and anchor the driveline to avoid trauma and potential infection. Any signs of infection, the implanting LVAD center should be notified urgently.

Percutaneous lead damage is a frequent cause for device exchange. The driveline should be inspected for tears in the outer casing or tears at the connecting sites, which can expose the internal wires. Repair of the driveline with safety clamps or reinforcement with tape may occasionally be needed, and should be performed only by a trained specialist and/or engineer. 

#### 5.1.2. Electrocardiogram

An ECG should be obtained to evaluate the heart rate and rhythm. A high index of suspicion is appropriate in patients presenting with vague symptoms since sustained ventricular tachycardia or ventricular fibrillation in a patient supported by a VAD may present with vague symptoms. Sustained (particularly incessant) ventricular arrhythmias generally precipitate worsening right-sided HF and, therefore, require prompt attention [31].

### 5.2. Device Interrogation

Shared care physicians who have undergone training (by the implanting LVAD team) and have the appropriate equipment may wish to interrogate the device controller for proper function, especially if there is any clinical change or suspicion of device malfunction. This is generally performed by the LVAD team, however. For all current devices, alarm alerts can be reviewed along with flow estimates, pump power, and pump speed. Normal ranges for device operating parameters have been described previously in this article. Alarms are displayed on the system controller display screen. The system controller also has audio warnings depending on the severity and type of alarm. When an alarm occurs, some information to help troubleshoot and resolve the issue are also displayed on the screen. Some examples of common system controller alarms are:

#### 5.2.1. HeartMate II and HeartMate 3

1. Hazard alarm: Flashing red heart or battery
  - “Low flow”
    The display will state to call the hospital contact. This warning suggests that the pump flow is estimated to be <2.5 Lpm. Ensure the driveline is intact, connections are secure, the controller is connected to a power source and the batteries have enough charge if the patient is on batteries. Assess the patient for causes of low flow such as hypertension (particularly in the case of HeartMate 3), significant volume loss, right-sided heart failure and pump thrombosis.
2. Advisory: Flashing yellow wrench or diamond
  This warning suggests that there is a mechanical, electrical or software issue with the system.

#### 5.2.2. Heart Ware

1. High priority: Flashing red triangle
  - “VAD stopped”
    The display will state to connect the driveline or change the controller. Ensure the driveline is intact, connections are secure, the controller is connected to a power source and the batteries have enough charge if the patient is on batteries. Assess for other causes of VAD dysfunction, including pump thrombosis.
  - “Critical battery”
    It indicates less than 5 min of battery remaining. Immediately replace the existing batteries with fully charged ones, or switch to the Power Module.
2. Medium priority: Flashing yellow triangle
  - “High watt”
    The display will state to call for medical assistance. This suggests that the pump has exceeded the high power alarm threshold. This could be due to a variety of causes, such as pump thrombosis, or an electrical malfunction and requires evaluation by a VAD specialist.
3. Low priority: Solid yellow triangle

If an LVAD patient presenting to a community-based practice or hospital is noted to have alerts or alarms, the implanting center should be notified. The LVAD team can assist with troubleshooting in case of an emergency. All patients are provided with contact information and on-call numbers for their respective center. Most centers also label the LVAD equipment with the on-call number. 

### 5.3. Diagnostic Laboratory and Imaging Tests

Laboratory tests that are particularly relevant for patients with an LVAD are evaluation for anemia, anticoagulation goals and hemolysis. Coumadin is recommended for all LVADs and thus the international normalized ratio (INR) should be checked frequently. Plasma-free hemoglobin >40 mg/dL is very specific for evidence of hemolysis; while lactate dehydrogenase (LDH) has greater sensitivity [35]. LDH 2.5× the upper limit of normal i.e., >600 IU/L for HeartMate II is highly suggestive of hemolysis. The cutoff value is less well defined for the centrifugal flow pumps, probably around 1.67× the upper limit of normal, i.e., >400 IU/L, or values significantly above baseline. Urinalysis for the presence of hemoglobinuria should also be performed. These should be checked when any hemodynamic derangement or LVAD malfunction is suspected. LDH is also generally checked periodically to screen for subclinical hemolysis. 

Echocardiography is crucial for the assessment of LVAD patients, including assessment of right and left ventricular function, filling pressures, inflow cannula position, orientation, and even obstruction, and adequacy of LV unloading. In addition, echocardiography can assess the presence and degree of aortic valve opening, which can aid in blood pressure assessment and interpretation. Periodic imaging at regular intervals is helpful for detecting complications such as aortic insufficiency. Echocardiography is also indicated if there are device alarms or signs or symptoms suggestive of LVAD dysfunction. Speed modifications with simultaneous echocardiographic imaging can help diagnose and troubleshoot various causes of LVAD dysfunction, as well as be useful for optimization of speed [36]. Guidelines for echocardiographic imaging of LVAD patients have also been published [37].

The LVAD inflow cannula and outflow graft can be extremely well imaged by contrast-enhanced gated CT scan. The images can be diagnostic for cannula malposition and outflow graft narrowing, kinking, or thrombosis [38]. Of note, CT scans of the chest performed for other indications, such as pulmonary embolism, may be misinterpreted as showing outflow graft thrombosis due to artifact. Therefore, dedicated imaging for LVAD evaluation at specialized centers should be pursued in case of uncertainty. Positron emission technology (PET) imaging with F-18 labeled fluoro-2-deoxyglucose can be helpful in identifying the presence and extent of infection in cases with suspected LVAD infection [39].

### 5.4. Medical Management 

Medical management of LVAD patients includes HF therapy, management of hypertension, and other concurrent conditions.

#### 5.4.1. Heart failure therapy

Components of HF therapy are similar to those used generally in systolic HF. Diuretics are used to treat volume overload. Angiotensin converting enzyme inhibitor or angiotensin II receptor blocker and/or beta blocker may be used to treat hypertension. Aldosterone antagonist may be used to reduce the need for potassium repletion in patients with preserved renal function. 

#### 5.4.2. Hypertension

For patients with CF-LVADs, the ISHLT guidelines recommend a mean arterial BP goal of ≤80 mmHg [31]. HF medications (angiotensin converting enzyme inhibitor, angiotensin II receptor blocker, beta blocker, hydralazine, nitrates) are the preferred agents for blood pressure management.

### 5.5. Device Therapy

The majority of the patients with an LVAD already have a pre-existing ICD. Concurrent ICD and LVAD therapy is feasible and safe, but the existing data on mortality benefit of ICDs in patients with CF-LVADs is inconsistent, with analysis of the UNOS registry data not showing a reduction in mortality with ICDs [40]. In patients without an existing ICD at the time of LVAD implant, most centers implant an ICD if there is a prior history of ventricular arrhythmias [41]. Also, ICD implantation in non-hospitalized patients awaiting transplantation is a class IIa recommendation [42]. There is no definite role for continuing cardiac resynchronization therapy post-LVAD [43].

The clinical benefit of a wireless implantable hemodynamic monitoring system (CardioMEMS) in patients with LVADs is being studied in the Intellect2 and HEMO-VAD studies [44,45]. Analysis of patients from the CHAMPION Trial who subsequently received LVADs showed that PA pressures decreased dramatically post-implant in all patients, but the degree of improvement was greater in the treatment group wherein physicians used the hemodynamic information to make management decisions [46]. This suggests that the CardioMEMS device may have clinical value in a certain group of LVAD patients who continue to experience heart failure symptoms.

### 5.6. Antithrombotic Therapy

Patients supported by LVADs should be treated with anticoagulant and antiplatelet agents to reduce the risk of thrombotic complications, such as device thrombosis and embolic stroke. Since these patients are also at risk for bleeding events, anticoagulation should be carefully monitored and adjusted. [31]. The device manufacturer recommends aspirin 81–100 mg for the HeartMate devices. For HVAD, the device manufacturer recommends daily aspirin dose > 81 mg, and in general, a daily dose of 325 mg. Recommendations from the HVAD manufacturers also include assessment of platelet inhibition and aspirin monotherapy adjusted accordingly or consideration of combination therapy such as aspirin 81 mg plus Aggrenox^®^ (aspirin plus extended-release dipyridamole) or daily aspirin 81 mg plus clopidogrel 75 mg. The goal INR in clinical trials and from the manufacturers for all the currents LVADs is 2.0 to 3.0 [31]. This may be adjusted by the LVAD center to a higher level in the setting of pump thrombosis history or hypercoaguable state and may be lowered in patients with a higher risk of bleeding (i.e., recurrent GI bleeding or a history of cerebral hemorrhage). With the decreased incidence of pump thrombosis with HeartMate 3, the safety of low-intensity anticoagulation targeting an INR between 1.5 to 1.9 is being tested. A pilot study suggested that this is achievable and safe with the HeartMate 3 LVAD [47] and a large-scale trial is warranted. 

Direct oral anticoagulants should be avoided in patients with LVADs. A higher rate of thromboembolic events on dabigatran led to early termination of a randomized controlled trial of dabigatran versus vitamin K antagonists in LVAD patients [48]. Low-molecular-weight heparin is used in some centers for bridging subtherapeutic INR in these patients. However, this should be done cautiously with close monitoring, since there appears to be an increased risk of major bleeding episodes with enoxaparin, and maybe even an increased risk of thromboembolic events [49].

## 6. Lifestyle Recommendations

Living with an LVAD requires adjustments to certain daily activities. Driveline care is of utmost importance. Since the source of LVAD infections is skin flora, ensuring sterility at the driveline exit site and aseptic precautions during dressing changes are essential for reducing the risk of infection. Measures such as ensuring hand hygiene, wearing sterile gloves, using antiseptic cleansing solutions around the driveline exit site, and use of sterile dressing material are important for prevention of infection. Many centers use standardized kits for inpatient dressing changes, as well as for home driveline maintenance. Patients and their caregivers are trained on driveline dressing change practices. Preventing wear and tear of the driveline, particularly at the exit site, and avoiding bends and kinks are also important to reduce the risk of infection. Patients are advised to limit exposing the driveline as much as possible. The number of people performing driveline care, dressings and assessment should be limited. Only the LVAD nurse/coordinator, surgeon if needed, and the patient or caregiver should assess the driveline and perform dressing changes. 

Patients should maintain a program of oral health care including regular professional care, the regular use of manual or powered toothbrushes, dental floss, and other plaque-removing devices to reduce the risk of infection.

Care should be taken during daily activities to avoid damage to external connections, for instance, patients may have to change the way they sleep so as to avoid lying on the LVAD and pulling the driveline or connections. Taking a shower is not permitted initially, but once the incisions are healed, it may be allowed at the discretion of the implanting center. A special shower bag is provided so the equipment does not get wet. Initially, post implant physical activity may be limited due to deconditioning; however, once stable, exercise as tolerated should be encouraged. The only restrictions include swimming and contact sports. 

Driving with an LVAD is safe for stable patients and can be resumed 3 months after LVAD implantation, once the incision has healed, after careful patient assessment. A survey of 390 LVAD patients showed that 90% felt that their driving capabilities were perfect or adequate [50]. Once the surgical incision is healed and the LVAD team deems the patient as stable, traveling is also permitted. During air travel, the security personnel will need to be notified that the patient has an LVAD and the need to carry LVAD equipment on the plane. Also, patients cannot go through metal detectors. Patients should be mindful of carrying all necessary equipment and spares and ensuring batteries are fully charged while traveling. An LVAD center should also be identified at the destination prior to travel, in case of emergency. Patients are permitted to have sex, but they should ensure there is no pressure on the equipment or excessive movement of the driveline at the exit site [51]. However, pregnancy is not advised as anticoagulation is more challenging to manage during pregnancy and patients are at high risk of cardiac complications and death. 

## 7. Procedures for Patients on LVAD Therapy

Patients with LVADs often require routine noncardiac procedures. These procedures should be carefully coordinated with an LVAD specialist, especially for recommendations regarding periprocedural management of antiplatelet therapy and anticoagulation. In addition, BP monitoring and management is paramount. There is an increased risk of bleeding, intraoperative hypotension, and a higher risk for acute kidney injury when these patients undergo noncardiac surgery [52]. However, patients can safely undergo major procedures such as joint replacements and vascular interventions. Major surgeries are recommended to be performed at LVAD centers. There are no specific guidelines for antibiotic prophylaxis prior to dental procedures. Many centers adopt the AHA guidelines for the prophylaxis of bacterial endocarditis for prosthetic heart valves, given the high frequency of infections and the potentially life-threatening consequences of infection [53]. Anticoagulation and antiplatelet therapy should not be discontinued for dental procedures. Certain dentists unfamiliar with LVAD patients may not be comfortable performing procedures on these patients. Finding a dentist experienced with LVAD patients is advisable, and procedures should be performed on a therapeutic INR. 

## 8. Complications of LVADs

While survival has improved with CF-LVADS, the adverse event profile remains high. At 1 month post-implant, nearly 30% and at 1 year, almost 80% of patients, require readmission [10]. The common LVAD-related complications, outside of the immediate post-operative period, are outlined in Table 4. Providers in the community may be the first medical contact for patients with LVADs experiencing a complication. Though management of these complications should be in concert with LVAD specialists, an awareness and understanding of the common complications can help with timely diagnosis and therapeutic intervention. 

### 8.1. LVAD Infections

Due to the presence of a percutaneous driveline exiting the skin, infections remain the Achilles heel of LVADs. Recent implantation of the first-in-man totally implantable LVAD aims to minimize this risk [54]. ISHLT classifies infections in LVAD patients as VAD-specific (involving the pump, cannula, pocket, or percutaneous driveline), VAD-related (infective endocarditis, blood stream infections including central venous catheter associated, mediastinitis) and non-VAD infections such as pneumonia [55]. 

Different techniques, largely limited to single-center experiences, have been shown to reduce infection rates and there is no universal protocol. Risk factors for driveline infection include trauma to the driveline exit site [56], younger age, higher body mass index and exposed driveline velour [57]. Meticulous exit site and driveline care and driveline immobilization are advised to reduce the risk of infection. 

Driveline drainage culture and blood cultures should be obtained, and imaging may be needed as well. It is important to note that driveline site assessment and obtaining specimens of the drainage should only be performed by members of the LVAD team (or designated shared care centers) in order to limit the number of times and the number of people to whom the driveline is exposed. Culture of any drainage is recommended. Abnormal swelling of the region surrounding the driveline site should be assessed by imaging studies (i.e., ultrasound, CT scan or PET scan) to assess the presence, extent, and appearance of any fluid collections [58]. Blood cultures are important to evaluate for occult bloodstream infections, because LVAD patients may not present with typical signs and symptoms. Typical pathogens are skin flora like coagulase-negative Staphylococci and *Staphylococcus aureus.* Gram-negative organisms like *Pseudomonas* spp. and Enterobacteriaceae, and fungal infections can also occur. Surgical evaluation for debridement of the driveline exit site may be needed depending on the extent of the infection, and should be performed by a surgical team with LVAD experience. Infectious diseases consultation may be needed, particularly in the case of polymicrobial infections or multidrug-resistant organisms. If bacteremia or sepsis are present, a chest and abdomen CT, transthoracic and even transesophageal echocardiogram should be obtained to evaluate for the source of the infection (especially in patients with pacemaker or defibrillator leads). There is an emerging role for PET imaging with F-18 labeled fluoro-2-deoxyglucose [39]. VAD infections are biofilm-based and difficult to eradicate. Treatment strategies for VAD infections include intravenous antibiotics followed by oral suppression in tandem with surgical debridement. Infections tend to be recurrent despite debridement, with Pseudomonas infection in particular, showing a lower success rate. The role of pump exchange is not clearly defined. One study noted a particularly poor prognosis with candidemia and concomitant presence of a cardiac-implanted electronic device, and failure to remove the device during pump exchange was associated with poor outcomes in 65% of patients. 

Some studies have shown that LVAD infections adversely affect survival after adjusting for age and comorbidities [59]. One prospective study showed a 22% overall infection rate of LVADs and a 5.6-times greater 1-year mortality in patients with infections [60]. Besides mortality, LVAD infections are associated with a greater risk of pump thrombosis, bleeding complications, prolonged hospitalization, need for pump exchange, and failure to achieve transplantation. Bloodstream infections, in particular, are associated with stroke and increased mortality [61]. Despite these risks, the presence of an LVAD infection has not been shown to have a negative impact on post-transplant outcomes [62]. 

### 8.2. Gastrointestinal (GI) Bleed

GI bleed is one of the most frequent complications after LVAD implant and the most common reason for hospital readmission. It is triggered by the use of antiplatelet and anticoagulant therapy. Additionally, CF-LVADs can cause acquired von Willebrand factor deficiency due to the high shear stress imposed by the LVAD motor, resulting in breakdown of large von Willebrand factor multimers. Also, continuous flow physiology and dysregulation of angiogenic factors have been shown to lead to development of arteriovenous malformations (AVMs) in the gastrointestinal tract, nasopharynx, brain, and other tissues [63]. 

Most patients present with melena, fatigue or dyspnea due to anemia or asymptomatic decline in hemoglobin picked up on routine evaluation. The initial assessment includes ensuring hemodynamic stability and transfusing blood products if needed (keeping in mind the potential for antibody sensitization in patients who are candidates for transplantation). Antiplatelet agents and anticoagulants are usually held and active reversal of INR is not needed, unless there is life-threatening hemorrhage, such as an intracranial hemorrhage. Decisions on blood transfusion and holding antiplatelet and antithrombotic agents should be made by the specialist LVAD team. Reversal of INR should only be performed by the specialist LVAD team. As the source of the GI bleed is most frequently AVMs, endoscopy is the diagnostic and therapeutic modality of choice. Endoscopic options for diagnosis include upper endoscopy, colonoscopy, video capsule endoscopy, and deep small bowel enteroscopy (e.g., single- and double-balloon enteroscopy). Since AVMs can be located throughout the GI tract, a combination of endoscopic techniques may be necessary. At many institutions, deep small bowel enteroscopy is the initial modality since AVMs are frequently located in the jejunum. The diagnostic and therapeutic yield of endoscopy remains high, with many patients undergoing repeated interventions [64]. For rapid bleeding, tagged RBC scan, CT angiography and interventional radiology procedures may be considered. 

Long-term pharmacologic therapy may be considered in select patients with recurrent GI bleeding. Drugs that are used are octreotide, a somatostatin analog, thalidomide, which is an antiangiogenic agent, and danazol [65]. In these patients, the INR goal is usually lowered and antiplatelet therapy may be decreased or discontinued altogether. 

### 8.3. Ventricular Arrhythmias

The reported prevalence of ventricular arrhythmias following LVAD implant varies from 22% to 59% [21]. They can occur even in patients with no prior history of ventricular arrhythmias. Arrhythmias in the immediate post-operative period are felt to be mediated by peri-operative adrenergic stimulation and the use of adrenergic agonists and positive cardiac remodeling on LVAD support leads to less arrhythmias later. Patients with a history of ventricular arrhythmias pre-LVAD are at increased risk even post-implant [66]. There is some data on the lack of beta blocker and ACE inhibitor use and etiology of cardiomyopathy as risk factors. 

Many LVAD patients can tolerate ventricular tachycardia (VT) or even ventricular fibrillation for hours or even days due to the continuous hemodynamic support provided by the LVAD, although some patients can present with “low flow” alarms due to an unsupported and failing right ventricle. In case of hemodynamic compromise, cardioversion can be safely performed, although external defibrillator pads should not be placed directly over the pump. Once the patient is stabilized, the causative mechanism for the arrhythmia should be evaluated. There can be several etiologic mechanisms, such as, suction events, myocardial scar related VT and apical inflow cannula related VT. A suction event occurs when the LV is excessively emptied, leading to myocardial tissue abutting the inflow cannula. This could be due to dehydration, extremely high speed or inflow cannula malposition [67]. In this scenario, LVAD interrogation could demonstrate low flow alarms and sustained speed drops. Echo with provocative maneuvers like Valsalva, can help demonstrate suction. Contrast enhanced gated CT timed for inflow cannula opacification can be helpful in demonstrating the inflow cannula position and may even demonstrate myocardial tissue abutting the cannula. Optimization of fluid status and/or decreasing pump speed may resolve VT caused due to suction. Antiarrhythmic medications and/or catheter ablation may be considered for myocardial scar and apical cannula related VT.

### 8.4. LVAD Malfunction 

LVAD malfunction due to technical failure is rare with the current generation of devices. These devices are much more durable than the prior pulsatile LVADs. The current devices can fail or malfunction due to tissue ingrowth or thrombus formation. Electric malfunction of internal components is rare, but can be life threatening. A technical fault with the external components, such as the controller, batteries, driveline and the connections can occur, and can, generally, be resolved by replacing the component. The initial evaluation involves assessing the hemodynamic stability of the patient. Auscultation, although limited, can help determine if the pump is on or off. Then ensure the device connections are secure, review LVAD function and alarms, ensure the batteries are charged or that the controller is connected to a power source. Assess the driveline for fractures and signs or wear and tear. The LVAD team should always be contacted if there is suspicion of an LVAD-related issue. Involving an LVAD technician or engineer can be very helpful. The device manufacturers can also be contacted via telephone. 

### 8.5. Pump Thrombosis

Thrombosis can occur in any component of the LVAD and patients are at risk of experiencing thromboembolic events such as stroke as well. Factors that predispose to the development of thrombosis are inadequate anticoagulation or antiplatelet therapy, interruption of anticoagulation, atrial fibrillation, concomitant infection, low pump speed and mechanical issues like stenosed or kinked outflow and inflow cannula malposition. HeartMate II has higher rates of device malfunction compared with HVAD (16.2% vs. 8.8%, respectively) [26] or HeartMate 3 (13.9% vs. 1.4%, respectively) [19]. A major advantage of HeartMate 3 is the significantly reduced risk of pump thrombosis. From 2011 to 2013, rates of thrombosis in HeartMate II were noted to increase from 2.2% at 3 months post-implant to 8.4% [68]. The PREVENT trial recommended an algorithmic clinical approach in HeartMate II patients, including implant technique, early post-operative initiation of anticoagulation with heparin bridging, pump speed optimization and maintenance of MAP ≤ 90 mmHg [69]. Following these clinical practices, the rate of confirmed pump thrombosis was found to be much lower at 4.8% at six months. Pump malfunction and stoppage as a result of pump thrombosis is associated with a high mortality. To avoid thrombosis, a target INR of 2.0 to 3.0 is recommended. Measurement of serial serum LDH levels is recommended to monitor for hemolysis.

Pump thrombosis should be suspected if there is evidence of hemolysis and/or suboptimal pump performance. Patients can present with asymptomatic hemolysis, hemoglobinuria, changes in LVAD performance like high power or low flow alarms, heart failure symptoms or even cardiogenic shock and multiorgan failure. Echocardiographic ramp studies [36] may be helpful in evaluating LVAD dysfunction related to thrombosis, wherein a lack of decline in the LV diastolic dimension with increasing pump speeds may be seen due to obstruction of the flow through the pump. CT angiography may be helpful in identifying an outflow graft thrombus. Management includes first stabilizing the patient. In case of pump thrombosis, resulting in pump stoppage, emergency expert medical care is required [31]. Definitive therapy for pump stoppage is surgical pump exchange. Administration of intravenous heparin, while adding a second antiplatelet agent and increasing the target INR range can be successful in managing late or chronic thrombosis. Certain centers have used thrombolytics like recombinant tissue-type plasminogen activator (rt-PA) or glycoprotein IIb/IIIa inhibitors in stable patients [70].

### 8.6. Neurological Emergencies

Neurologic complications are the leading cause of mortality, accounting for 19% of deaths following CF-LVAD implant, primarily from hemorrhagic and ischemic strokes [10]. Most ischemic strokes in LVAD patients are cardioembolic in origin [71]. Factors that increase risk of stroke are ones that promote thrombosis, such as stasis due to obstruction in the pump inflow or outflow, inadequate anticoagulation, and coexistent infection. Strict BP management is essential to reduce the risk of stroke, particularly hemorrhagic stroke, as suggested by the ENDURANCE Supplemental Trial data. 

In the ENDURANCE Trial, the HVAD cohort had a higher incidence of stroke (29.7% vs. 12.1%) at 2 years post-implant [26]. Post hoc analysis showed a MAP ≥ 90 mmHg was associated with stroke. Other possible factors associated with stroke could have been the pump inflow design (absence of sintering on the inflow cannula in the initial pumps), lower INR in HVAD patients, and lower-dose aspirin use. Subsequently, in the ENDURANCE Supplemental Trial, with strict blood pressure management, there was a nonsignificant reduction in the overall stroke rate at 12 months, from 22.3% in ENDURANCE to 16.9% in the Supplemental Trial, with a 50% reduction in the hemorrhagic stroke rate [34]. The MOMENTUM3 Trial also showed a reduction in the rate of strokes at 2 years with HeartMate 3 compared to HeartMate II (9.9% vs. 19.4%) [19]. A direct comparison of HeartMate 3 with HVAD has not been performed.

Early diagnosis and immediate assessment are critical, and if applicable, the stroke team should be activated, anticoagulation held and CT head without contrast should be performed emergently. In case of hemorrhagic stroke, neurosurgical consultation should be obtained and reversal of anticoagulation with vitamin K, fresh frozen plasma and/or prothrombin protein complex should be considered in consultation with an LVAD specialist. In case of suspected ischemic/embolic stroke, CT angiogram of the head and neck should be performed and endovascular thrombectomy should be pursued if patients are candidates. Hemorrhagic strokes can also occur as a result of rupture of mycotic aneurysms, which are known to be associated with bacteremia associated with LVAD or driveline infection [61]. 

### 8.7. Heart Failure

The vast majority of VADs are isolated to the LV due to poor outcomes in those that need biventricular VAD support. Thus, one can have continued heart failure post-LVAD due to either right ventricular (RV) failure or due to inadequate unloading of the LV by the device or biventricular failure. Acute or early RV failure post-LVAD implantation has a reported incidence of 10% to 44% and can lead to longer hospital length of stay and higher perioperative mortality [72,73]. Even 1-year survival for patients with early RV failure is worse than those without RV failure; 59% vs. 78% (*p* < 0.001), based on data from the HeartMate II BTT Trial [74]. Late or chronic RV failure, occurring a few months after LVAD implant is a distinct entity, though there is no standardized definition for this entity yet. Prolonged RV failure is associated with worse outcomes, not just long-term survival, but also quality of life, functional capacity, and increased hospitalizations and resource utilization [72]. The pathophysiology for RV failure post-LVAD is complex, and involves an interplay of increased preload to the RV following unloading of the LV with an LVAD, in the setting of pre-existing RV dysfunction. Also, the implantation of an LVAD, particularly the intra-pericardial pumps, distorts the RV geometry and displaces the interventricular septum, reducing the contribution of the septum to the RV contractility. 

When a patient with an LVAD presents with heart failure, assessment should be directed towards gauging whether there are right-sided signs like elevated jugular venous pressure, ascites, pedal edema or left-sided findings like orthopnea, paroxysmal nocturnal dyspnea, or both. Echocardiogram and invasive hemodynamic assessment should be obtained to confirm the diagnosis. In RV failure, echocardiogram will generally reveal a small, underfilled LV, enlarged RV with reduced systolic function, dilated inferior vena cava, tricuspid regurgitation. Right heart catheterization findings include elevated right atrial pressure > 18 mmHg, low pulmonary capillary wedge pressure ≤ 18 mmHg and a low cardiac index < 2.0 L/min/m^2^. LVAD interrogation may demonstrate low flow. Acute RV failure is managed with diuretics, inotropes, pulmonary vasodilators and even a temporary right ventricular assist device in severe cases. Chronic RV failure management is challenging, and management is focused on diuresis, and consideration for heart transplantation if the patient is a candidate. Decreasing the LVAD speed may help. Palliative care consultation may be beneficial in refractory cases, who are not eligible for transplant. 

In patients presenting with left-sided heart failure, assess for hypertension, LVAD dysfunction (due to pump thrombosis or technical fault), aortic insufficiency and inadequate LV unloading. Treatment is directed towards the underlying etiology. Systemic vasodilators are preferred for treatment of hypertension. Management of aortic insufficiency is challenging: diuresis, inotropic support and LVAD speed increase may help temporize the situation. Transcatheter aortic valve replacement has been performed at a few centers with mixed results. If volume overload is due to inadequate LV unloading, a dilated LV, significant mitral regurgitation, aortic valve opening with each beat can be seen on echocardiogram. Increasing the LVAD speed may result in resolution of the symptoms. 

## 9. Emergency Care 

In the event of an emergency, patients with LVADs should be taken to their respective VAD center. If a patient with an LVAD is brought to a non-VAD center, it is imperative that emergency medical services providers also bring all the LVAD-related equipment, including back-up and spare components (the batteries, controllers, power module), since it will be necessary to support the patient and may be helpful in troubleshooting the issue. If the patient is being transported to a non-VAD center, a potential VAD center should be immediately notified for medical assistance and potential transfer as soon as possible. The patient’s LVAD center and personnel should also be promptly notified. Oftentimes, family members of patients with an LVAD are trained in emergency procedures, and if feasible, they should be transported with the patient. Code status information, particularly in patients with a DT LVAD, should be identified and the patient’s wishes respected. 

### Unresponsive Patients and Cardiopulmonary Resuscitation

Care for an unresponsive LVAD patient is challenging due to several different reasons. Their unique physiology alters basic physical examination findings, which makes assessing their perfusion status difficult. In addition, assessing the LVAD for function is essential and consideration of LVAD-related complications as possible etiologic mechanisms for the patient’s deterioration is needed. Also, there has been debate in the past regarding the role for chest compressions in patients with an LVAD, which has led to varying and, on occasion, conflicting instructions being given to personnel involved in emergency care. 

Guidelines for cardiopulmonary resuscitation (CPR) in adults with LVADs were published in 2017 by the American Heart Association (AHA) [75]. For the first responder, the basic approach is analogous to the basic CPR principles. An airway, breathing, circulation (ABC) approach to CPR is recommended.

- Assess responsiveness. If there is no response, call for help.
- Check if the patient is breathing and assist ventilation as needed with supplemental oxygen, airway adjuncts and intubation, as deemed necessary. End-tidal CO_2_ should be monitored.
- Determine if adequate perfusion is being maintained by assessing mental status, skin color, temperature and capillary refill. It is important to remember that LVAD supported patients may not have a palpable pulse or recognizable BP with an automatic machine, even with adequate perfusion.
- Assess if the LVAD is working by auscultating for an LVAD hum over the precordium. If there is a mechanical hum, then the device is likely working effectively. Ensure connections to the controller are secure, and ensure power supply is adequate.
- If the VAD is not functioning, and MAP is ≤ 50 mmHg, then chest compressions and rescue breaths should be initiated per the basic life support protocol. If a connection issue is identified, then the connection should be reestablished, provided the period of LVAD discontinuation is known to be less than 30 minutes. If there has been a prolonged period of LVAD discontinuation, thrombus formation is likely and restarting the device could result in a fatal embolism.
- If the LVAD is not functioning, and no connection problems are identified, a system controller change-out may be required. This should only be done by a trained provider or caregiver. Family members are trained to make this controller change.
- If the LVAD is still not functioning, chest compressions should be continued, defibrillation and medications should be administered per the advanced cardiac life support algorithms. Temporary mechanical support, including extracorporeal membrane oxygenation may be needed.
- If the LVAD is functioning, but the patient is unconscious with MAP ≤ 50 mmHg or P_ETCO2_ < 20 mmHg, external chest compressions are indicated. If the LVAD is functioning, only gentle compressions are required, as only RV compressions are needed (the LVAD will continue to provide systemic flow). With an arterial line, the compressions can be adjusted as needed to maintain a perfusing arterial pressure. Overly vigorous chest compressions should be avoided, particularly in the early postoperative phase, as there is risk of dislodging the device and causing myocardial injury and hemorrhage. Defibrillation and medications should be administered per the advanced cardiac life support algorithms.
- If the LVAD is functioning and both respirations and perfusion appear adequate and the patient is unconscious, assess for causes of unconsciousness like stroke, coma (e.g., hypo- or hyperglycemia), or sedation.
- It is reasonable to provide standard post-cardiac arrest care, including targeted temperature management and early percutaneous coronary intervention, when indicated. Of note, patients with an LVAD need adequate anticoagulation, which may be difficult to monitor during therapeutic hypothermia.

**Figure 3:**
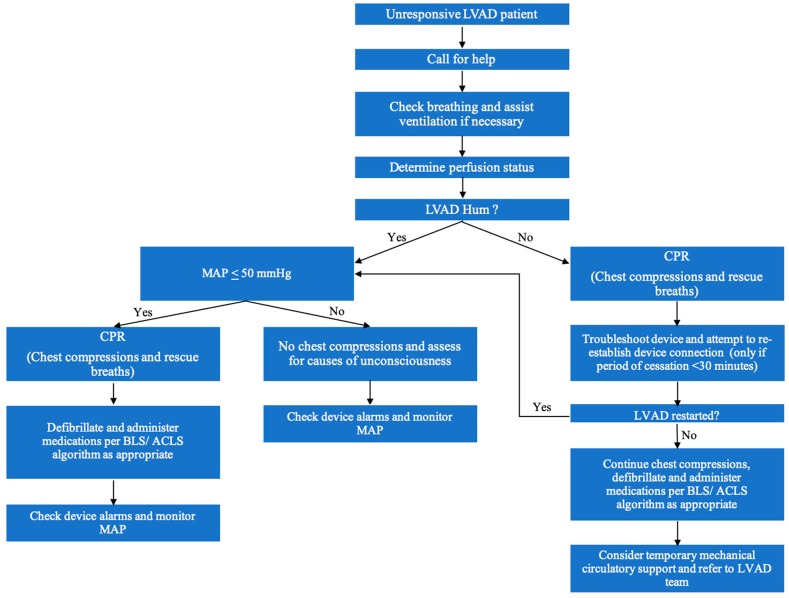
Approach to an unconscious patient with an LVAD.

In all hypotensive patients with an LVAD, an arterial catheter should be placed as soon as possible in either the radial or femoral position. Ultrasound guidance should be used. Continuous BP monitoring is essential as it is the only reliable means to monitor for adequate perfusion in these patients. Approach to the management of an unresponsive LVAD patient is depicted in the form of a flowchart in Figure 3.

If available, point of care echocardiography should be performed. It is non-invasive, easy and quick to perform and can provide real-time information about the LVAD performance and complications like RV dysfunction, suction and cardiac tamponade. 

## 10. Conclusions

The number of ambulatory LVAD patients is rising and their life expectancy is improving, as well owing to improvements in technology and management. Non-LVAD specialists are increasingly encountering LVAD patients in a variety of clinical settings and, therefore, should be well prepared to perform the initial assessment and provide management for these complex patients. Also, as this therapy becomes more widely available, and adverse events reduce, joint management between the implanting centers and providers in the community will also become increasingly prevalent. 

## Figures and Tables

**Table 1:** Typical LVAD operating parameters.

Pump Parameter	HeartMate II	HeartMate 3	HVAD
Typical speed, rpm	8800–10,000	5000–6000	2400–3200
Speed adjustment increment, rpm/increment	200	100	20
Flow, L/min	4–7	4–6	4–6
Power, Watts	5–8	4.5–6.5	3–7
Pulsatility index (or HVAD, peak to trough)	5–8	3.5–5.5	2–4 L/min/beat

**Table 2:** Major Differences Among the Current LVADs.

Feature	Heartmate II	HVAD and HeartMate 3
Pump design	Axial flow	Centrifugal flow
Size and surgical implant	Large Surgical implantation in a pre-peritoneal pocket	Small and compact Implanted directly adjacent to the heart in the pericardial space
Blood flow and power consumption		Relationship between blood flow and power consumption is more linear Flow and cardiac output estimation more accurate Patient’s hematocrit is also used to estimate the serum viscosity, which is used in the flow calculation
Hydrodynamic performance (determined by the relation between the flow rate and pressure head i.e., the differential pressure between the inlet in the left ventricle and the outlet in the aorta [13,14])	Less change in flow for a given change in pressure gradient across the pump (i.e., afterload minus preload) Flow is less pulsatile	Larger change in flow for a given change in pressure gradient across the pump (i.e., afterload minus preload) Results in phasic changes in LVAD flow during systole and diastole as the pressure gradient changes during the cardiac cycle Greater pulsatility Since the pressure gradient across the pump is determined by the systemic blood pressure, strict blood pressure management is imperative for normal pump function.
Additional feature		Algorithm which periodically modulates the pump speed Termed “Artificial Pulse” for HeartMate 3 and “Lavare Cycle” for HVAD Promotes washing of the pump and facilitates intermittent opening of the native aortic May reduce rates of pump thrombosis with HeartMate 3, and aortic valve insufficiency and stroke with HVAD [15,16].

**Table 3:** Summary of key LVAD clinical trials.

Study, Year (Reference)	N	Device Tested	Indication	Design	Patient Population	Outcome
REMATCH, 2001 **[6]**	129	HeartMate XVE	DT	Prospective 1:1 HeartMate XVE vs. medical therapy	NYHA functional class IV for 60 days, LVEF < 25%, and peak VO2 < 14 mL/min/kg (unless on balloon pump, IV inotropes, or physically unable to perform exercise test), or intra-aortic balloon pump (IABP) or IV inotrope dependent for 14 days	1- and 2-yr HeartMate XVE survival of 52% and 23% vs. 25% and 8% on medical therapy
INTREPID, 2007 **[20]**	55	Novacor	DT	Prospective nonrandomized	Inotrope-dependent patients	1-yr Novacor survival of 27% vs. 11% on medical therapy
HeartMate II, 2007 **[21]**	133	HeartMate II	BTT	Prospective nonrandomized	Transplant candidates with systolic HF and NYHA functional class IV and inotrope dependence or need for IABP support	75% survival to transplant, recovery, or ongoing support although remaining eligible for transplant at 6 months
HeartMate II, 2009 **[22]**	192	HeartMate II	DT	Prospective randomized 2:1 HeartMate II vs. HeartMate XVE	NYHA functional class IIIB or IV symptoms for >45 of the last 60 days, LVEF<25%, and peak VO2 <14 mL/min/kg (unless on IABP, IV inotropes, or physically unable to perform exercise test), or IABP dependent for 7 days or IV inotrope dependent for 14 days	1- and 2-yr HeartMate II survival of 68% and 58% vs. 55% and 24% with HeartMate XVE
HeartMate II post-approval, 2011 **[23]**	169	HeartMate II	BTT	Prospective nonrandomized	Consecutive patients eligible for transplant in INTERMACS	90% survival to transplant, recovery, or ongoing support at 6 months
HeartMate II post-approval, 2014 **[24]**	247	HeartMate II	DT	Prospective nonrandomized	Consecutive patients eligible for DT in INTERMACS	1- and 2-yr survival of 74% and 61%
ADVANCE, 2012 **[25]**	137	HVAD	BTT	Prospective nonrandomized. HVAD compared with 499 patients who received FDA-approved LVADs in INTERMACS	Transplant candidates	90.7% survival to transplant, recovery, or ongoing support on the original device vs. 90.1% in control group at 6 months
ENDURANCE, 2017 **[26]**	446	HVAD	DT	Prospective, DT patients randomized 2:1 HVAD vs. HeartMate II	Chronic, advanced HF, NYHA functional class IIIB or IV despite recommended medical therapy, EF< 25%, and ineligible for transplantation at the time of enrollment	- HVAD noninferior to HeartMate II with respect to survival free from disabling stroke or device removal for malfunction or failure. - More device malfunction or device failure requiring replacement in HeartMate II (16.2% vs. 8.8%). - More strokes in HVAD (29.7% vs. 12.1%).
MOMENTUM 3 long-term cohort, 2018 **[27]**	366	HeartMate 3	BTT, DT and bridge to candidacy	Prospective, randomized, 1:1 HeartMate 3 vs. HeartMate II. Pre-specified interim analysis at 2 years	Advanced heart failure requiring LVAD. 60% ineligible for transplantation. 85% on IV inotropic therapy	Survival free from disabling stroke or reoperation to replace/remove a malfunctioning device at 24 months, in 79.5% of HeartMate 3 vs. 60.2% of HeartMate II (*p* < 0.001 for superiority).
MOMENTUM 3 full cohort, 2019 **[19]**	1028	HeartMate 3	BTT, DT and bridge to candidacy	Prospective, randomized, 1:1 HeartMate 3 vs. HeartMate II. Adaptive trial design. Follow up period 2 years.	Advanced heart failure requiring LVAD. 61% were ineligible for transplantation. 86% were on intravenous inotrope therapy.	- Survival at 2 years free of disabling stroke or reoperation to replace or remove a malfunctioning device 74.7% vs. 60.6% (*p* < 0.01 for superiority). - 96.9% freedom from pump exchange. - For every 100 patients implanted with HeartMate 3 rather than HeartMate II: - 22 pump thrombosis events averted, 20 strokes averted, 68 bleeding events averted (36 gastrointestinal) - Reduction in cardiac arrhythmias, particularly ventricular arrhythmias. - Reduction in readmissions and days spent in the hospital.

**Table 4:** Summary of common LVAD-related complications.

Complication	Management Strategy
LVAD infections	Obtain local wound and blood cultures Imaging if evaluation is suspicious for driveline infection or if bacteremia is present Antibiotics and possible surgical debridement
Bleeding (non-surgical)	Hold aspirin and warfarin For severe bleeding, consider reversal of anticoagulation after consultation with an LVAD specialist Transfuse if needed Tagged RBC scan or interventional procedure if rapid bleeding Upper GI source suspected–consider small bowel enteroscopy Lower GI source suspected–consider colonoscopy
Ventricular arrhythmias	Consider anti-arrhythmic medications Cardioversion if hemodynamically unstable Optimize fluid status
LVAD malfunction	Ensure device connections are secure Review LVAD alarms Evaluate LVAD flow and patient stability
Pump thrombosis	Assess LVAD flow and patient stability Early pump thrombosis generally requires pump exchange. Augment anticoagulation and antiplatelet therapy
Neurologic complications (stroke, intracranial hemorrhage)	Activate stroke team Obtain CT head. Hold anticoagulation until imaging is performed. Ischemic stroke–Interventional radiology procedure if patient is a candidate. Otherwise, antiplatelet and anticoagulation in consultation with stroke team and LVAD specialist. Hemorrhagic stroke–Neurosurgical consultation and reversal of anticoagulation
Heart failure	Assess for right-sided versus left-sided HF from incomplete unloading Optimize LVAD speed and diuresis
